# HEPBURN - investigating the efficacy and safety of nebulized heparin versus placebo in burn patients with inhalation trauma: study protocol for a multi-center randomized controlled trial

**DOI:** 10.1186/1745-6215-15-91

**Published:** 2014-03-25

**Authors:** Gerie J Glas, Johannes Muller, Jan M Binnekade, Berry Cleffken, Kirsten Colpaert, Barry Dixon, Nicole P Juffermans, Paul Knape, Marcel M Levi, Bert G Loef, David P Mackie, Manu Malbrain, Marcus J Schultz, Koenraad F van der Sluijs

**Affiliations:** 1 Laboratory of Experimental Intensive Care and Anesthesiology (L · E · I C · A), Department of Intensive Care Medicine, Academic Medical Center, M0-210, Meibergdreef 9, 1105 AZ Amsterdam, the Netherlands; 2 Department of Intensive Care Medicine, Academic Medical Center, Amsterdam, the Netherlands; 3 Department of Intensive Care, University Hospital Gasthuisberg, Leuven, Belgium; 4 Department of Intensive Care, Maasstad Hospital, Rotterdam, the Netherlands; 5 Department of Intensive Care, Ghent University Hospital, Ghent, Belgium; 6 Department of Intensive Care, St Vincent’s Hospital, Melbourne, Australia; 7 Department of Intensive Care, Red Cross Hospital, Beverwijk, the Netherlands; 8 Department of Internal Medicine, Academic Medical Center, Amsterdam, the Netherlands; 9 Department of Intensive Care, Martini Hospital, Groningen, the Netherlands; 10 Department of Intensive Care, Ziekenhuis Netwerk Antwerpen - Stuivenberg, Antwerp, Belgium

**Keywords:** Inhalation trauma, Pulmonary coagulopathy, Nebulization, Heparin, Mechanical ventilation

## Abstract

**Background:**

Pulmonary coagulopathy is a hallmark of lung injury following inhalation trauma. Locally applied heparin attenuates lung injury in animal models of smoke inhalation. Whether local treatment with heparin benefits patients with inhalation trauma is uncertain. The present trial aims at comparing a strategy using frequent nebulizations of heparin with standard care in intubated and ventilated burn patients with bronchoscopically confirmed inhalation trauma.

**Methods:**

The Randomized Controlled Trial Investigating the Efficacy and Safety of Nebulized HEParin versus Placebo in BURN Patients with Inhalation Trauma (HEPBURN) is an international multi-center, double-blind, placebo-controlled, two-arm study. One hundred and sixteen intubated and ventilated burn patients with confirmed inhalation trauma are randomized to nebulizations of heparin (the nebulized heparin strategy) or nebulizations of normal saline (the control strategy) every four hours for 14 days or until extubation, whichever comes first. The primary endpoint is the number of ventilator-free days, defined as days alive and breathing without assistance during the first 28 days, if the period of unassisted breathing lasts for at least 24 consecutive hours.

**Discussion:**

As far as the authors know, HEPBURN is the first randomized, placebo-controlled trial, powered to investigate whether local treatment with heparin shortens duration of ventilation of intubated and ventilated burn patients with inhalation trauma.

**Trial registration:**

NCT01773083 (http://www.clinicaltrials.gov), registered on 16 January 2013.

Recruiting. Randomisation commenced on 1 January 2014.

## Background

Inhalation trauma adds to the morbidity and mortality of burn patients [1,2]. Indeed, up to one third of burn patients require hospitalization due to inhalation trauma and a substantial percentage of these patients develop respiratory insufficiency. Moreover, approximately one third of burn patients with inhalation trauma eventually die, markedly more than burn patients without pulmonary involvement.

The underlying pathophysiology of inhalation trauma is only partly understood. Injury is thought to be at least the result of heat exposure to the airway mucosa or inhalation of noxious particles or toxic gases [3]. Injury of airway mucosa could induce an inflammatory response resulting in vascular leakage and pulmonary edema [4]. Pulmonary cast-formation could certainly play a role, since one study of burn patients shows disturbed alveolar fibrin turnover [5], and several preclinical studies show obstructive airway casts after smoke inhalation [6-9].

At present, therapy for inhalation trauma is merely supportive [10]. Animal models of smoke injury [6,7,9] and clinical trials of burn patients with inhalation trauma suggest that local treatment with heparin has beneficial effects [11,12]. The objective of the present trial is to determine the clinical efficacy and safety of frequent nebulizations of heparin in burn patients with inhalation trauma. Furthermore, the present trial investigates local effects of heparin on pulmonary coagulation and inflammation.

## Methods

### Design

The ‘Randomized Controlled Trial Investigating the Efficacy and Safety of Nebulized HEParin versus Placebo in BURN Patients with Inhalation Trauma’ (HEPBURN) is an international multi-center, double-blind, placebo-controlled, two-arm study. The trial is conducted in accordance with the declaration of Helsinki [13]. The Institutional Review Board of the Academic Medical Center, Amsterdam, the Netherlands, approved the trial protocol. The trial is registered at http://www.clinicaltrials.gov (NCT01773083). Patients or their legal representative have to give written informed consent before any study-related procedure is performed.

### CONSORT diagram

**Figure 1:**
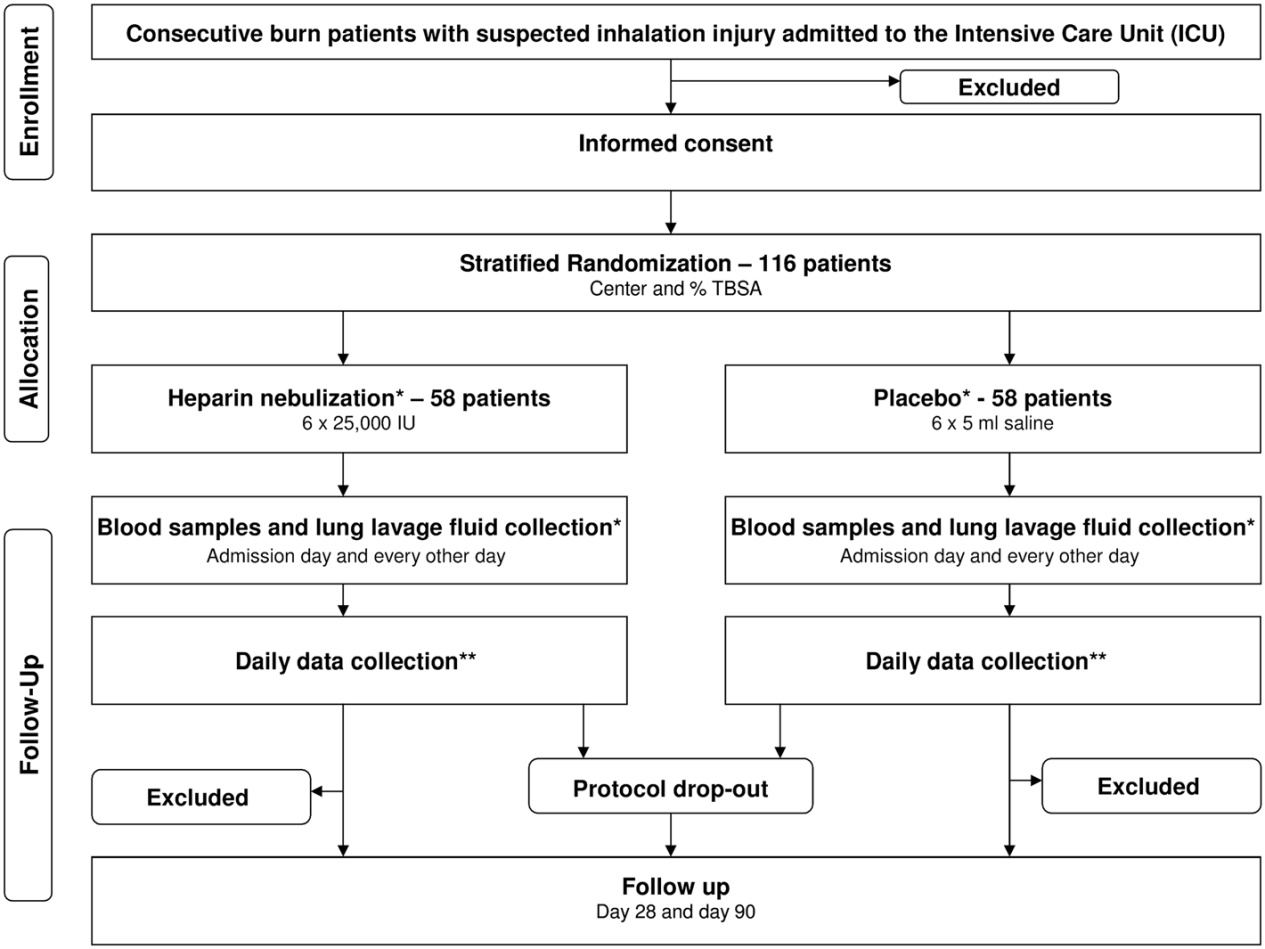
**CONSORT diagram of HEPBURN.** *Until successful liberation from mechanical ventilation or for the maximum duration of 14 days. **For the maximum duration of 28 days.

The CONSORT [14] diagram of HEPBURN study is presented in Figure 1. Consecutive burn patients with suspected inhalation trauma are screened. Demographic data and data on the extent of injury are registered regardless of meeting enrollment criteria. If excluded from participation, the reason(s) for exclusion are registered. For screening of inhalation trauma, a clinical [15] and a bronchoscopic [16] scoring system are used (Table 1).

**Table 1:** Clinical scoring and grading of inhalation trauma

A. Clinical scoring system [15]		
1	History of being trapped in a house or industrial fire in an enclosed space	
2	Production of carbonaceous sputum	
3	Peri-oral facial burns affecting nose, lips, mouth, or throat	
4	Altered level of consciousness at any time after the incident and including confusion	
5	Symptoms of respiratory distress, including a sense of suffocation, choking, breathlessness, and wheezing or discomfort affecting the eyes or throat, indicating irritation of the mucous membranes	
6	Signs of respiratory distress, including stertorous, labored breathing, and tachypnea or auscultatory abnormalities, including crepitations or rhonchi	
7	Hoarseness or loss of voice	
B. Severity of inhalation trauma [16]		
0	No injury	Absence of carbonaceous deposits, erythema, edema, bronchorrhea, or obstruction
1	Mild injury	Minor patchy areas of erythema or carbonaceous deposits in proximal or distal bronchi
2	Moderate injury	Moderate degree of erythema, carbonaceous deposits, bronchorrhea or bronchial obstruction
3	Severe injury	Severe inflammation with friability, copious carbonaceous deposits, bronchorrhea or obstruction
4	Massive injury	Evidence of mucosal sloughing, necrosis or endoluminal obliteration

A. Clinical scoring system [15]. One point is given for the presence of each clinical factor considered to be suggestive of smoke inhalation to a total of seven points; a score of greater than 2 is considered fulfilling the criteria for unequivocal smoke inhalation. B. Severity of inhalation trauma based on bronchoscopic findings.

### Setting

HEPBURN is performed in three centers specialized in care of burn patients in the Netherlands (Red Cross Hospital, Beverwijk; Martini Hospital, Groningen; Maasstad Hospital, Rotterdam) and three specialized centers in Belgium (University Hospital Gasthuisberg - Leuven; Ziekenhuis Netwerk Antwerpen (ZNA) - Hospital Network Antwerp; Ghent University Hospital, Ghent).

### Study population

Intubated and ventilated burn patients, > 18 years of age with bronchoscopically confirmed inhalation trauma are eligible for participation. Notably, intubated and ventilated patients without skin burns, but with bronchoscopically confirmed inhalation trauma, are also eligible for participation in HEPBURN.

Patients are excluded if they are not enrolled within the first 36 hours after trauma, if intubated and ventilated for longer than 24 hours prior to trial enrollment, and if unlikely to survive for more than 72 hours or have burns covering > 60% of total body surface area (TBSA). Patients are also excluded if the expected duration of intubation and ventilation is less than 24 hours. Other exclusion criteria are history of severe chronic obstructive pulmonary disease (COPD) with (non-invasive) ventilation or oxygen therapy at home or repeated systemic corticosteroid therapy for acute exacerbations, witnessed or bronchoscopically proven aspiration, any history of pulmonary hemorrhage in the past three months, any history of clinically important bleeding disorder, known allergy to heparin or heparin-induced thrombocytopenia, and pregnancy or breast feeding.

### Randomization, blinding and intervention

Randomization is performed using ALEA® software (TenALEA consortium, Amsterdam, the Netherlands) and uses random blocks. Prior to randomization, patients are stratified for burn center and TBSA < 20% versus TBSA ≥ 20%. Patients are identified by study ID-numbers (randomization numbers) that are linked to the assigned study medication (heparin or normal saline). The pharmacist at each study site has access to the confidential file containing the randomization scheme, which links ID-numbers to appropriate ampoules. Since heparin and placebo are packed identically, they are provided with a temporary additional label ‘*verum*’ in case of heparin, or ‘placebo’ in case of saline. Before dispensing the medication to the ward, this additional label will be removed by the pharmacy, after which the medication is blinded for the study team. Nebulized volumes are identical.

Patients are randomly assigned to nebulization of study medication consisting of unfractionated sodium heparin (Pfizer, Melbourne, Australia) or sodium chloride 0.9% (Pfizer, Melbourne, Australia), administered every four hours (that is six times per day). For this, the total volume of identically packed 5 mL ampoules containing 25,000 IU of unfractionated heparin or 0.9% NaCl, is nebulized using an AeronebPro system (Aerogen Inc, Sunnyvale, CA, USA), connected to the ventilator circuit closest to the patient, but always in between the patient and the heat and moist exchanger if used. To prevent potential damage from nebulized heparin to the expiratory valve of the ventilator, a filter is placed in the expiratory limb of the circuit. Each nebulization session lasts 15 minutes. Nebulization of study medication is continued until tracheal extubation or death, but for a maximum duration of 14 days.

### Protocol drop-out

Nebulization of study medication can be discontinued temporarily when heparin-induced thrombocytopenia (HIT) is suspected. Nebulization of study medication is definitely discontinued when HIT is confirmed. Furthermore, nebulization of study medication can be discontinued temporarily in case of surgical procedures, clinically relevant bleeding (mandating infusion of clotting factors, fresh frozen plasma or platelet concentrates), activated partial thromboplastin time of > 150 seconds and a platelet count of ≤ 10 × 10^9^/L. Finally, study medication can be discontinued temporarily with appearance of excessive blood in sputum or lavage fluids. Timing of re-start is left to the discretion of the attending physician, but it is stressed to re-start study medication as soon as it is considered safe. In case of any life-threatening bleeding event, protamine sulphate can be used as an *antidotum* to heparin, at the discretion of the attending physician.

### Concomitant medication

Intravenous or oral anticoagulant therapy, if indicated, is allowed. Routine use of N-acetylcysteine or any other mucolytic agent, either nebulized or directly installed into the airways, is not allowed. They should only be used when viscous mucus is considered problematic, at the discretion of the attending physician.

### Standard procedures

Attending physicians are advised to use lung-protective ventilation strategies, including the use of lower tidal volumes (≤ 6 mL/kg predicted body weight) or lower airway pressures (≤ 30 cmH_2_O). Levels of positive end-expiratory pressure (PEEP) and inspired oxygen (FiO_2_) are titrated on PaO_2_, preferably using a PEEP/FiO_2_ table, and according to local guidelines. If spontaneous ventilation is well tolerated, it is used from then till the end of ventilation. Thereafter, weaning from ventilation is performed by stepwise lowering of pressure-support level, or by spontaneous breathing trials, according to local guidelines. As soon as patients are ready to be weaned from the ventilator, the pressure-support level is lowered stepwise to 5 cmH_2_O. In case of spontaneous breathing trials, the patient’s ability to breathe spontaneously is verified for 30 minutes using a T-piece or continuous positive airway pressure. Patients are assessed daily if they are ready to be tracheally extubated. Extubation criteria are as follows: patient is responsive and cooperative, has an adequate oxygenation (saturation > 90%) with PaO_2_/FiO_2_ of 150 mmHg or more and FiO_2_ of 40% or less, is hemodynamically stable with no uncontrolled arrhythmia, and has a rectal temperature > 36.0°C. Attending physicians are advised not to perform tracheotomies in the first 14 days after inclusion. If a patient meets extubation criteria, but remains intubated because of planned surgeries requiring general anesthesia, this is reported.

Analgo-sedation and fluid management are according to local guidelines. Analgo-sedation is achieved by continuous or bolus infusion of either fentanyl or morphine in combination with a benzodiazepine or propofol, titrated to individual needs. Procedure-related analgesia and sedation are at the discretion of the attending physician. Sedation depth is measured on a daily basis, and is used to titrate infusions of sedatives.

In general, patients are resuscitated using buffered hypertonic or isotonic saline solutions, targeting a urine output of 0.5 to 1.0 ml/kg/h. If necessary to maintain serum albumin concentration > 15 g/L, human albumin is administered. After the first 24 hours, fluid administration is dictated by clinical needs.

Antibiotic prophylaxis and thromboprophylaxis are applied in accordance to local guidelines. We chose to allow the use of systemic or oral anticoagulants. Although we expect that the use of (intravenous) anticoagulant agents and prophylactic antibiotics will be similar in both groups, we will record the use of these agents.

### Follow-up

On the day of ICU admission, demographic and baseline data, as well as data on disease severity and extent of burn injury are collected. Data collection includes: gender, age, height, weight, functional status (independent, partially dependent or totally dependent), cardiac status (heart failure, according to the New York Heart Association (NYHA) [17], acute coronary syndrome *or* persistent ventricular tachyarrhythmia), alcohol status (in the past two weeks: 0 to 2 drinks/day, or more than 2 drinks/day), smoking status (in the past three months: never, former, or current). Data collection on relevant medical history include: COPD for which inhalation therapy or oral steroids are used, respiratory infection in the last month (upper or lower), history of active cancer, weight of loss more than 10% in the last six months, history of diabetes mellitus, chronic kidney disease, liver failure (Child-Pugh classification) [18], acquired immunodeficiency syndrome and use of relevant medications (including oral anti-diabetics, antibiotics in the last three months, statins, and corticosteroids). Regarding extent of burn injury, collected data include TBSA, partial or full thickness burn, Belgian Outcome in Burn Injury Study Group Index [19] and the Abbreviated Burn Severity Index (16).

Data on standard of care and clinical outcome variables (described below) are collected on a daily basis for the maximum duration of 28 days, until first discharge from ICU or death (whichever comes first). Data on length of stay in ICU and - hospital, mortality in ICU and hospital, and re-admissions to ICU are assessed on days 28 and 90 (Table 2).

**Table 2:** Time schedule of study procedures

	**Day of admission (day 1)**	**Every day**^**b/c**^	**Days 1, 3, 5, 7, 9, 11 and 13**^**c**^	**Day of hospital discharge**	**Day 28**	**Day 90**
Screening and randomization						
screening^a^	X					
demographic data	X					
inclusion criteria	X					
exclusion criteria	X					
informed consent	X					
randomization	X					
Before start study medication						
blood sampling	X					
non directed broncho-alveolar lavage sampling	X					
Clinical procedures						
chest X-ray		X				
nebulization of study medication^c,d^		X				
blood sampling^c^			X			
non-directed broncho-alveolar lavage sampling^c^			X			
Clinical data collection						
%TBSA	X					
SAPS II	X					
Karnofsky score	X					
ABSI	X					
SOFA score		X				
LIS/OI		X				
ventilatory settings		X				
relevant medications^e^		X				
sedation		X				
fluid balance		X				
Other data collection						
adverse event		X				
use of blood products		X				
use of protamine		X				
use of n-acetylcysteine		X				
use of vasopressors^f^		X				
bronchoscopy^g^		X				
pneumonia (VAP)		X				
date of hospital discharge				X		
Survival, if not:				X	X	X
date of death						
cause of death						

^a^Of all patients with suspected inhalation injury.

^b^For the maximum duration of 28 days.

^c^For the maximum duration of 14 days or until the patient is successfully weaned from mechanical ventilation.

^d^If temporarily or definitely discontinued, reason should be registered.

^e^Such as antibiotics, immunosuppressives, systemic anticoagulants.

^f^Such as dopamine, dobutamine, norepinephrine, or epinephrine.

^g^To remove foreign particles and accumulated secretions.

The following variables are collected daily: ventilator freedom (described below), requirement for non-invasive mechanical ventilation (if yes: indication and duration per day), Oxygenation Index (OI) [20], Sequential Organ Failure Assessment (SOFA) scores [21], Lung Injury Score (LIS, based on chest X-ray findings, PaO_2_/FiO_2,_ PEEP level and respiratory compliance) [22], presence of ventilator-associated pneumonia (VAP), presence of acute respiratory distress syndrome (ARDS) [23], number of bronchoscopy-guided cleaning attempts per day to clean the larger airways, and use of relevant medications (including antimicrobial agents, immunosuppressive agents, and any anticoagulant agent).

The following laboratory data are collected daily: glycemia, urea, creatinine, aspartate aminotransferase, alanine aminotransferase, bilirubin, hemoglobin (Hb), platelets, prothrombin time (PT), aPTT, white blood cells count, albumin, and arterial blood gas analysis.

Typical ICU-related therapy variables to collect daily include: ventilation variables, need for lung rescue therapy (such as prone ventilation, extracorporeal membrane oxygenation or oscillator), analgo-sedation variables (including sedations scores, using for example, the Richmond Agitation Sedation Scale (RASS) [24], and delirium scores, using for example, the Confusion Assessment Method (CAM-ICU) [25], pain score using, for example, a 10-point score, cumulative dose of sedatives and analgesics (including fentanyl, morphine, midazolam, and propofol), and fluid resuscitation variables including cumulative fluid balance [26], amount and type of infused fluids (for example, crystalloids, colloids and albumin), and daily amount of enteral/parenteral nutrition [27]. After the first 14 days, data on tracheotomies are collected.

Any surgical procedure, the amount of blood loss, presence of serious bleedings (defined as any clinically important bleeding from the lung (prompting bronchoscopy) or any clinically important bleeding requiring surgery or transfusion of clotting factors or blood products (red cells, platelets, plasma) is reported. Type and number of units transfused are specified as well as reasons to (temporarily) discontinue study medication, as described above, are reported as well.

### Study endpoints

The primary endpoint - is the number of ventilator-free days, defined as the number of days alive and breathing without the assistance of a ventilator during the first 28 days. Thus, a patient must be free of ventilation for 24 hours to have one ventilator-free day. If, after successful extubation the patient requires re-intubation and ventilation due to a surgical procedure, this episode will not count as a ventilator day. However, if after surgery, ventilation is prolonged due to respiratory insufficiency, the day(s) will be counted as ventilator days.

Secondary endpoints - are subdivided into clinical and laboratory outcome variables and safety variables. Clinical outcome variables include: (a) length of stay (ICU and hospital); (b) mortality (28- and 90-day); (c) mortality (ICU and hospital); (d) daily LIS; (e) daily OI; (f) daily SOFA scores; (g) cumulative dosages of sedatives; (h) incidence of VAP; (i) total number of bronchoscopy-guided cleanings of the larger airway. Laboratory outcome variables include: (a) levels of markers of coagulation and fibrinolysis in blood and lung lavage fluid, including but not limited to tissue factor, activated factor VII, antithrombin, thrombin-antithrombin complexes, activated protein C, plasminogen activator activity, tissue plasminogen activator, urokinase plasminogen activator, plasminogen activator inhibitor 1, fibrin degradation products and plasma levels of heparin; (b) levels of markers of inflammation in blood and lung lavage fluid, including but not limited to IL-1beta, IL-6, IL-8, IL-10, TNF-alpha, growth factors and markers of complement activation; and (c) biological markers of lung injury in blood and lung lavage fluid, including but not restricted to total protein, albumin and IgM, Clara Cell 16 protein and soluble receptor for advanced glycation endproducts. Safety variables include (a) occurrence of relevant bleedings - requiring surgery, requiring transfusion of clotting factors, requiring transfusion of blood products (red cells or platelets or plasma), any clinically important bleeding from the lung (prompting bronchoscopy); (b) any other transfusion of blood products - red cells or platelets or plasma; (c) confirmed HIT; and (d) prolonged activated partial thromboplastin time (aPTT > 150 seconds).

### Collection of blood samples and lung lavage fluid

In selected centers, blood is drawn through the existing arterial line and processed immediately. A total volume of 20 mL is drawn every other day for a maximum period of 14 days, and always before performing the lung lavage. A non-directed lung lavage is performed every other day for a maximum period of 14 days or less if the patients are successfully weaned from mechanical ventilation. Lung lavage is performed as described before [28]. In brief, 20 ml sterile 0.9% saline is instilled via a standard 50 cm 14 gauge tracheal suction catheter. The distal end of the catheter is introduced via the endotracheal tube and advanced until resistance is encountered. Immediately after instillation of the saline solution over 4 to 5 seconds, fluid is aspirated before withdrawal of the catheter. Plasma and lung lavage fluid are centrifuged and stored at −80°C until assays (described above) are performed.

### Statistical considerations

Sample size calculation - in a previous trial testing the efficacy of nebulized heparin, critically ill patients requiring ventilation for at least 48 hours showed a reduction of 4.6 days of ventilation (mean (SD) 22.6 (4.0) versus mean (SD) 18.0 (7.1)) [29]. We conservatively estimate that the improvement in burn patients with inhalation trauma is less than in a heterogeneous group of critically ill non-burn patients. We consider a reduction of three days of ventilation clinically relevant. We require 58 patients per treatment arm to observe a difference in the number of ventilator-free days at day 28 with (α < 0.05 at 80% power). Although burn patients with inhalation trauma usually have fewer ventilator-free days than critically ill non-burn patients, the standard deviation appears to be similar between burn injury patients [30] and critically ill patients in general [31], indicating that the sample size of 58 per arm for a total of 116 is sufficient to observe an improvement of three ventilator-free days (nQuery Advisor Software version 7.0, Statistical Solutions Ltd, Cork, Ireland).

Stratification - considering the fact that TBSA is associated with prolonged duration of ventilation [32], we want to avoid an uneven distribution of the severity of burns between study groups. Therefore, patients are stratified based on TBSA (≥ 20% versus < 20%). Provided that this stratification will only be used to ensure an equal distribution, and stratification is based on the median TBSA, it does not affect the sample size of this study.

Interim analysis - blind interim analysis for safety is performed after 58 patients are successfully included and reached the endpoint. Suspected unexpected serious adverse events (SUSARs) and serious adverse events (SAEs) (any death; all serious bleedings requiring surgery, transfusion of clotting factors or blood products; any clinically important bleeding from the lung; requirement of transfusion of blood products; and confirmed heparin-induced thrombocytopenia) that are possibly related to study medication will be compared using the Chi-square test. If the data safety monitoring board (DSMB) (see below) has concern regarding the incidence of serious adverse events, it can request unblinded data. The investigators will remain blinded for the groups until the study is completed. If complications occur significantly more often in the intervention group (*P* ≤ 0.05), the study is terminated due to harm.

### Statistical analysis

Patient characteristics will be compared and described by appropriate statistics. Data will be analyzed on an intention-to-treat basis. Normally distributed variables will be expressed by their mean and standard deviation and non-normally distributed variables will be expressed by their medians and 95% confidence levels. Categorical variables will be expressed as N (%). To test for normality, a D’Agostino and Pearson omnibus test will be performed [33]. Differences between groups will be tested by Student’s *t*-test for normally distributed data and Mann–Whitney *U*-test for not normally distributed data. Categorical variables will be compared with the Chi-square test or Fisher’s exact tests or when appropriate as relative risks. The difference of the primary effect variables will be analyzed using the Mann–Whitney *U*-test. The rate of freedom from ventilation will be analyzed according to the Kaplan-Meier method [34] and the results will be compared with the log-rank test hazard ratio’s are reported with 95% confidence limits. To take into account the stratification factors, we will perform an adjusted analysis. TBSA levels are group level data that will be included as a second level covariate in a multilevel linear regression model to assess the adjusted effect of our randomized treatment. Also the treatment effect on coagulation markers is investigated. Because they are clustered within each patient (repeated measurements) we will also use a multilevel model to evaluate the influence of the cluster effect on the model fit, that is we will compare the model fit without a second level (the patient) with the model fit that includes the patient as a second level. When appropriate, statistical uncertainty will be expressed by the 95% confidence levels. Statistical significance is considered to be at a *P* of 0.05. Analysis will be performed with SPSS version 18.1 (SPSS Inc., Chicago, IL, USA).

### Study organization

The Steering Committee is composed of the principal investigators of the participating centers who contribute to the design and revisions of the study protocol. The national coordinators in the Netherlands and in Belgium ensure that all local necessary ethical and regulatory approvals are obtained before start of patient inclusion. In individual participating centers, local coordinators provide scientific and structural leadership in their center. They guarantee the integrity of data collection and ensure timely completion of case report forms (CRFs). The trial coordinator trains and monitors the participating centers to ensure the study is conducted according to ICH-GCP guidelines [35], guarantees the integrity of data collection and ensures timely completion of CRFs. An independent monitor performs study monitoring. The independent DSMB is composed of four independent persons (Prof. Dr. Marcelo Gama de Abreu, Prof. Dr. Samir Jaber, Prof. Dr. Paolo Pelosi, and Prof. Dr. Antonio Artigas Raventós). The DSMB reviews the overall status of the program (number of patients enrolled overall and in each center, adherence to the protocol overall and by each center) and receives an interim analysis on safety (described below). The DSMB will meet by conference calls. The first meeting is scheduled after 15 patients are enrolled. Subsequent to this meeting, the DSMB will meet every six months.

All adverse events reported are sent to the DSMB for review in a blinded fashion. All (possibly) related and unrelated severe adverse events (SAEs) and suspected unexpected serious adverse reactions (SUSARs) will be sent to the DSMB within 24 hours after being received by the coordinating center. Non-serious study-related adverse events will be sent every week. Non-serious study-unrelated adverse events are recorded in an overview list (line-listing), submitted once every half year.

If the DSMB has concern regarding the incidence of SAEs, the DSMB can request and will be supplied with unblinded data. The investigators will never see these data. When complications occur significantly more often in the intervention group (*P* ≤ 0.05), terminating the study due to harm will be considered.

## Discussion

Pulmonary coagulopathy and subsequent fibrin deposition is an important feature of a wide variety of inflammatory lung conditions. Indeed, pulmonary disturbances in coagulation and fibrinolysis have been described with remarkable uniformity with the acute respiratory distress syndrome (ARDS) [36-38], pneumonia [36,39], and ventilator-associated lung injury [40]. In addition, pulmonary coagulopathy has been observed with chronic inflammatory conditions of the lung, such as asthma, [41], COPD [42], idiopathic pulmonary fibrosis [43] and interstitial lung diseases [44,45].

More recently we showed that local coagulopathy is also a feature of inhalation trauma [5]. The presence of airway obstructive casts composed of mucus, epithelial and inflammatory cells and fibrin, is considered a major factor that contribute to pulmonary dysfunction in patients with inhalational trauma [46]. In patients who need ventilatory support, overdistention of aerated lung parts may occur while other parts remain atelectatic due to airways obstructed by casts [11,46].

Coagulation and inflammation are intricately related processes that considerably affect each other [47]. There are several pathways by which procoagulant and inflammatory mechanisms can induce or worsen lung injury [48]. Activation of coagulation is both a consequence and a contributor to ongoing lung injury [49]. Given the procoagulant shift in the pulmonary compartment in patients with inhalation trauma [5], pulmonary coagulopathy could be considered as a potential therapeutic target.

Heparin is widely used as an anticoagulant and exerts its effect through its ability to increase the activity of antithrombin III, thereby reducing thrombin generation [50]. Notably, the biological action of heparin extends beyond its anticoagulant activity [29]. First, heparin has been shown to inhibit leukocyte activation, which may have an impact on pulmonary inflammation [31]. Second, heparin inhibits fibroblast proliferation and collagen deposition [51] and may thus affect tissue remodeling. Third, heparin has been shown to reduce adhesion of bacteria and viruses, which may have an impact on infectious complications in mechanically ventilated patients [52]. Finally, heparin may interfere with the recruitment of neutrophils towards inflammatory sites [53-55].

Beneficial effects of heparin nebulization alone or in combination with other agents are demonstrated in several preclinical models of inhalation trauma [6,7,9,11,12]. In an ovine model of smoke inhalation injury followed by sepsis after *Pseudomonas aeruginosa* instillation in the lungs, nebulized heparin results in improved oxygenation and reduces airway obstruction by casts [9]. Beneficial effects are also seen with systemically administered heparin, but only at relatively high dosages [56].

Two retrospective clinical studies with nebulized heparin in patients with smoke inhalation injury at least suggest that local heparin treatment could have beneficial effects [11,12]. It is uncertain whether this treatment truly benefits patients with inhalation trauma, however. First, the two clinical studies so far tested the efficacy of heparin in combination with mucolytic agents and bronchodilators. Second, these trials have some methodological problems, since they were single-center trials using historical controls. Third, it is uncertain whether local treatment with heparin is safe [11,12,57]. As far as the authors know, HEPBURN is the first double-blind, randomized, placebo-controlled trial of nebulized heparin in burn patients with inhalation trauma, focusing both on clinical efficacy and safety. In addition to clinical outcomes, HEPBURN is designed to determine whether nebulized heparin reduces pulmonary coagulopathy and inflammation. In a model of lung injury, the LPS-induced procoagulant state in the lungs could be reversed through aerosol delivery of anticoagulants [58]. In this model, heparin also reduced inflammatory cell numbers in BAL fluid, suggesting that nebulized heparin could also impact pulmonary inflammation. It remains uncertain, however, whether beneficial effects of nebulized heparin depend on possible effects on pulmonary inflammation, or solely on effects on pulmonary coagulation. To gain more insight into this, we will compare patients who receive heparin with patients who receive placebo with respect to several inflammation and coagulation variables in lung lavage fluid. This may enable us to identify a possible mechanism by which nebulized heparin affects the course of inhalation injury in burn patients.

Studies in healthy volunteers demonstrate that approximately 8% of nebulized heparin reaches the lower respiratory tract, of which 40% remains present 24 hours after inhalation [59]. The dose of nebulized heparin (applied as a single dose) that results in measurable increases in plasma aPTT is 150,000 IU and the half-life of the inhaled heparin is estimated to be 28 hours [60]. Since the type of nebulizer could affect the percentage of dose reaching the lower respiratory tract, we use the same nebulizer type as in previous clinical trials of nebulized heparin in critically ill patients [29,61]. In this trial, both dosing and duration of local heparin therapy are different from the two retrospective clinical studies of nebulized heparin in burn patients [11,12]. We based the dose, frequency and duration of heparin nebulization on a previous trial of critically ill patients, in which heparin nebulization was safe and associated with an increase in the number of ventilator-free days [29].

A major concern regarding safety could be the occurrence of serious bleedings. It might be argued that, due to endothelial injury and the increased vascular permeability resulting from the inhalational injury, the systemic impact of nebulized heparin and the risk of pulmonary bleedings might be higher in burn patients with inhalation injury when compared to critically ill patients without inhalation injury. However, two preclinical studies of burn and smoke inhalation injury models reported that heparin nebulization did not affect the systemic clotting time [6,9]. To date, data on safety of local heparin treatment in burn patients with inhalation trauma are limited. HEPBURN stops if complications occur significantly more often in one arm.

We chose the number of ventilator-free days at day 28 as the primary endpoint of this trial, a clinically relevant outcome parameter in critically ill patients. The effect of heparin may vary depending on the severity of injury [6]. However, the sample size of this trial does not allow subgroup analyses, for example, the evaluation as to what extent the efficacy of heparin depends on the severity of burn injuries.

To increase uniformity between participating centers, mechanical ventilation and weaning are performed according to our study protocol. Attending physicians are also advised not to use a tracheotomy in the first 14 days after inclusion and routine use of mucolytic agents is not allowed. In addition, data on applied care and on the use of relevant concomitant medications such as anticoagulants and mucolytics are collected. Nevertheless, differences in care practices amongst the different participating centers may be considered as a confounding factor in our study. On the other hand, it may also indicate whether nebulized heparin is applicable even when there are (subtle) differences in standardized care.

In conclusion, as far as the authors know, the HEPBURN study is the first prospective double-blind, randomized, placebo-controlled trial powered to test the hypothesis whether nebulized heparin increases the number of ventilator-free days in burn patients with inhalation trauma, to evaluate the safety of this strategy, and to investigate the local effects of heparin on pulmonary coagulopathy and inflammation.

## Trial status

Patient recruitment started in October 2013.

## Abbreviations

ABSI: Abbreviated Burn Severity Index; AE: adverse event; AR, adverse reaction; APC: activated protein C; aPTT: activated partial thromboplastin time; ARDS: acute respiratory distress syndrome; AT: antithrombin; BAL: bronchoalveolar lavage; CAM-ICU: Confusion Assessment Method; CC16: Clara cell protein; CDC: Centers for Disease Control and Prevention; COPD: chronic obstructive pulmonary disease; CPAP: continuous positive airway pressure; CRF: case report form; DSMB: Data Safety Monitoring Board; EudraCT: European drug regulatory affairs Clinical Trials; FDP: fibrin degradation products; FVIIa: activated factor VII; GCP: good clinical practice; HIT: heparin-induced thrombocytopenia; IL: interleukin; IMP: investigational medicinal product; IMPD: investigational medicinal product dossier; LIS: Lung Injury Score; LPS: lipopolysaccharide; LOS: length-of-stay; NBL: non-directed broncho-alveolar lavage; NYHA: New York Heart Association; OI: Oxygenation Index; PAA: plasminogen activator activity; PAI: plasminogen activator inhibitor; PEEP: positive end-expiratory pressure; PSV: pressure-support ventilation; PT: prothrombin time; RASS: Richmond Agitation Sedation Scale; SAE: serious adverse event; SAPS: simplified acute physiology score; SBT: spontaneous breathing trial; SOFA: Sequential Organ Failure Assessment; sRAGE: soluble receptor for advanced glycation endproducts; SUSAR: suspected unexpected serious adverse reaction; TATc: thrombin antithrombin complex; TBSA: total body surface area; TF: tissue factor; TNF-alpha: tumor necrosis factor-alpha; tPA: tissue plasminogen activator; uPA: urokinase plasminogen activator; VAP: ventilator-associated pneumonia; ZNA: Ziekenhuis Netwerk Antwerpen

## Competing interests

The authors declare that they have no competing interests.

## Author’s contributions

GG, KvdS, JB and MJS designed the study. JB performed the power calculation and designed the statistical analysis plan. GG prepared the initial draft of this manuscript. JM, BC, KC, BD, NJ, PK, ML, BL, DM, MM approved study design. All approved the initially submitted version of this manuscript. All approved the revised and final version of this manuscript.
